# Predicting the HIV/AIDS Knowledge among the Adolescent and Young Adult Population in Peru: Application of Quasi-Binomial Logistic Regression and Machine Learning Algorithms

**DOI:** 10.3390/ijerph20075318

**Published:** 2023-03-30

**Authors:** Alejandro Aybar-Flores, Alvaro Talavera, Elizabeth Espinoza-Portilla

**Affiliations:** 1 Department of Engineering, Universidad del Pacífico, Lima 15072, Peru; a.aybarf@up.edu.pe (A.A.-F.); ag.talaveral@up.edu.pe (A.T.); 2 Faculty of Health Sciences, School of Medicine, Universidad Continental, Lima 15046, Peru

**Keywords:** HIV/AIDS knowledge, adolescents and young adults, health structural determinants, quasi-binomial logistic regression, machine learning

## Abstract

Inadequate knowledge is one of the principal obstacles for preventing HIV/AIDS spread. Worldwide, it is reported that adolescents and young people have a higher vulnerability of being infected. Thus, the need to understand youths’ knowledge towards HIV/AIDS becomes crucial. This study aimed to identify the determinants and develop a predictive model to estimate HIV/AIDS knowledge among this target population in Peru. Data from the 2019 DHS Survey were used. The software RStudio and RapidMiner were used for quasi-binomial logistic regression and computational model building, respectively. Five classification algorithms were considered for model development and their performance was assessed using accuracy, sensitivity, specificity, FPR, FNR, Cohen’s kappa, F1 score and AUC. The results revealed an association between 14 socio-demographic, economic and health factors and HIV/AIDS knowledge. The accuracy levels were estimated between 59.47 and 64.30%, with the random forest model showing the best performance (64.30%). Additionally, the best classifier showed that the gender of the respondent, area of residence, wealth index, region of residence, interviewee’s age, highest educational level, ethnic self-perception, having heard about HIV/AIDS in the past, the performance of an HIV/AIDS screening test and mass media access have a major influence on HIV/AIDS knowledge prediction. The results suggest the usefulness of the associations found and the random forest model as a predictor of knowledge of HIV/AIDS and may aid policy makers to guide and reinforce the planning and implementation of healthcare strategies.

## 1. Introduction

Acquired Immunodeficiency Syndrome (AIDS), caused by the Human Immunodeficiency Virus (HIV), is one of the most devastating infectious diseases in human history since its discovery in 1981: approximately 78 million people have been infected and some 35 million individuals have died from HIV/AIDS-associated diseases since the beginning of the epidemic worldwide [1]. These facts, combined with the governmental action of each nation, have determined the worrying variety of scenarios that have characterized the problem that this disease represents and the need to study its nature and spread [2,3].

The term “AIDS” refers to a set of symptoms that occur in the final stage of an infection caused by the Human Immunodeficiency Virus. In the same perspective, HIV is a virus that attacks immune cells called CD4 cells, which are a type of T-cell [4]. When HIV attacks and infiltrates these cells, it reduces the body’s ability to fight other diseases. It is transmitted by contact with certain body fluids of an individual infected with HIV (blood, semen, vaginal fluid, anal mucus and breast milk), most commonly during unprotected sex or by sharing contaminated needles [4]. On the other hand, Sims [4] states that AIDS occurs when the virus has destroyed the immune system, leaving the patient highly susceptible to other life-threatening infections. Without treatment, an HIV infection is likely to develop into AIDS as the immune system gradually weakens. Nonetheless, advances in antiretroviral therapy (ART) mean that a decreasing number of individuals are progressing to this stage: antiretroviral drugs can help patients reduce their viral load, improving their quality of life and prolonging their life expectancy [4].

However, despite advances in the field of health sciences over the past twenty years, namely, the reduction in the number of new HIV infections worldwide from 3.4 million in 1996 to 1.5 million in 2021 and 650,000 people dying of HIV-related causes in 2021 [5,6], HIV and AIDS continue to be a health threat for numerous countries around the world, with implications for the design and implementation of public policies and for the day-to-day development of various population groups at risk. Through the adverse economic and social consequences of HIV/AIDS on individuals and households, the epidemic also creates challenges for social policy in the form of a diminished state [7]. Furthermore, the current service coverage is still inadequate, and the pace of its expansion is too slow to meet global targets (by 2021, there were 38.4 million people globally living with HIV, and about 5.9 million people did not know that they were living with HIV) [8]. Success in the global HIV response is not evenly or equitably distributed (human rights violations, along with widespread gender-based violence, stigmatization and discrimination, continue to obstruct access to health services, particularly for children, adolescents, young women and vulnerable populations such as LGBT communities, among others) in some countries and regions [9].

Nowadays, the response against HIV/AIDS is considered a crucial and priority matter within public health policies globally due to the devastating effects of the disease for its high potential for spread and lethality in the absence of treatment and countermeasures [10]. In that sense, spreading knowledge and awareness about HIV/AIDS results in one of the key strategies used in the prevention and control of the epidemic worldwide. Inadequate knowledge and risky practices are the main obstacles in preventing the spread of the virus [11]. In many countries, sexually transmitted infections (STIs) and unplanned pregnancies are frequently observed among adolescents. Young people started having sex with one or multiple sexual partners indiscriminately, and this facilitated the spread of STIs and HIV. Therefore, adolescents in general are at an increased risk of contracting HIV through sexual transmission. Thus, the need to understand the knowledge and attitude of young people towards HIV/AIDS and public efforts for a personalized approach to disease control and prevention through key education and awareness programs becomes even more important [12]. Successful disease-control efforts depend on understanding both the distribution and frequency of health behaviors and measuring the general public’s knowledge of HIV/AIDS and the associations of their knowledge and attitudes with different socio-demographic factors [12].

Faced with this, the epidemiological analysis of infections and diseases (which translates into prospective studies, trends in the evolution and patterns of infection dynamics and influences that various indicators have on these) allows for better decision-making at a governmental level in public/social health and the actions that derive from the evaluations of the results are established as the main axes of the efforts against the epidemic and its ravages [9]. In the case of HIV/AIDS studies, mathematical modeling and computational simulations of epidemiological infection-control efforts have become imperative tools for the evaluation of different policies at the state level, of the evolution of health at the population level and of interventions in governmental sectors related to health that have generated promising results in recent decades [13].

Under these circumstances, artificial intelligence has the potential to improve clinical care, including HIV care, by optimizing HIV/AIDS diagnosis, treatment selection and risk stratification for prevention strategies [14]. Artificial intelligence (i.e., ML) has been introduced into the healthcare field as a means of improving the exactness and accuracy while reducing the number of time-consuming tasks that require human intervention [15]. Because of its ease of use, this innovation could provide a useful tool, allowing for quicker intervention [15]. Given the advances in the scientific understanding of HIV diagnosis and treatment, novel strategies are urgently needed to prevent new HIV infections [14]. These models in the medical industry have an immense capacity to develop diagnostic and prognosis indicator applications that can aid in the proper initial treatment of life-threatening diseases, such as HIV/AIDS [16,17]. In this sense, artificial intelligence for HIV prevention has been applied by using machine learning to identify people who might benefit from HIV testing, pre-exposure prophylaxis or other risk-reduction strategies [14], including studies from the USA [18,19,20,21,22], Denmark [23], and eastern Africa [24]. In addition, natural language processing is a potential strategy for optimizing future tools including electronic health records to identify patients who might benefit from pre-exposure prophylaxis or other medications, although the benefits of predictive performance will need to be evaluated against the additional computational resources required [14]. Moreover, random forest machine learning algorithms were applied to predict virologic outcomes among HIV infected adults in countries such as Switzerland using electronically monitored combined antiretroviral treatment adherence [25].

In that regard, the major contributions that the current research has provided to the existing literature may prove useful and relevant to policy makers and health promoters working in the Peruvian government. First, it has described the characteristics of adolescents and young adults (our target population) in Peru according to their knowledge about HIV/AIDS. Second, this study has pinpointed the structural determinants of health (demographic, economic and social factors) in the Peruvian territory that have an empirical influence on the knowledge about HIV/AIDS among the target population. Finally, this research has established the machine learning model that provided the best goodness-of-fit and accuracy for the classification of the HIV/AIDS knowledge in the youth population in Peru by comparing parametric and non-parametric estimation techniques. Thus, our study could aid the design and management of public health policies in Peru since, through the evaluation and monitoring of infection at the population level, it is possible to analyze the trends that the disease has adopted and the factors that influence it in order to choose strategies and measures to control and, subsequently, eradicate it in the long term [26].

## 2. Literature Review

Currently, the fight against HIV/AIDS is considered a crucial and priority aspect within public health policies worldwide because of the devastating effects of the disease due to its potential for spread and the high cost of health services associated with this infectious disease [9]. This urge to analyze the epidemiological situation of HIV/AIDS has been addressed through previous research that has been conducted in this regard, as the disease evolves at different levels of study approach [2,3].

### 2.1. Current Status of Health Efforts against HIV/AIDS in Peru

According to the National Center for Epidemiology, Disease Prevention and Control, Ministry of Health of Peru (CDC-Peru) [27], since 1983 when the first AIDS case was reported in the country, up to November, 2022, a total of 158,134 cases of HIV infection have been reported, of which 49,001 are in the AIDS stage. In the same sense, CDC-Peru [27] reports that in 2021 the ratio is 4:1 men to women in diagnosed cases of HIV infection and for AIDS cases it is 3–4 men to one woman. On the other hand, CDC-Peru [27] points out that regarding the reported HIV cases in the period from 2018 to November 2022 the most frequent route of transmission is the sexual route with 95%, followed by 0.4% by mother–child transmission (vertical) and 0.1% parenteral route. In turn, CDC-Peru [27] indicates that—during the period 2018–November 2022—the majority of HIV cases (45%) were reported from Lima, the capital of the country, followed by Loreto (7%), La Libertad (6%), Callao (5%), Ucayali (5%), Piura (4%) and Arequipa (3%); for AIDS cases reported, 76% of them are concentrated in four regions: Lima, Junín, Callao and La Libertad.

In this context, the Office of the People’s Advocate [28] states that, although there is recognition of the actions taken so far by the state, the balance of the multisectoral response to the HIV/AIDS epidemic in the country shows the absence of clear policies from the different sectors of the state. The Office of the People’s Advocate [28] states that several evaluations and reports recognize the need for an organic multisectoral response in the fight against AIDS in order to optimize the use of resources and enhance the actions of the different actors. It also points out that, at present, the immediate response to HIV in the country is determined, in an important way, by the commitments assumed with the Global Fund for the execution of projects submitted and financed. This certainly implies great opportunities, linked to the magnitude of funding and its potential impact if well conducted, but it also involves great threats linked to the possibility of not taking advantage of this investment if it is used in ineffective or poorly implemented interventions [28]. Although it is the health sector that has shown the greatest progress, the Office of the People’s Advocate [28] states that it has not yet been possible to mobilize an effective comprehensive response to the HIV and AIDS epidemic from this sector, in which the requirements of the affected population to access better living conditions and enjoy comprehensive healthcare have become increasingly evident.

### 2.2. Association between Structural Determinants of Health and HIV/AIDS

HIV/AIDS and its evolution and treatment, evaluated from a biomedical and public health policy framework, are influenced by multifactorial determinants. Ama et al. [29] point out that these cofactors can be referred to as social determinants of health and can be defined as economic, social, cultural, psychological, and environmental/biological conditions that influence health. Likewise, these social determinants have an impact on layers: from individual determinants (not modifiable) to macro determinants (involving economic, cultural and environmental conditions) [30,31].

Thus, researchers’ interest in understanding the reasons why people with different socioeconomic characteristics experience health and disease differently has led the debate to a new approach that recognizes that the health status of the subject is determined by social behavioral and structural factors [32].

According to the findings of the scientific literature, several authors have established connections of different cuts between health determinants and HIV prevalence: variation and change in macroeconomic factors may slow down HIV proliferation in the developing world [33], low levels of average monetary income are related to higher HIV incidence rates [34], the association between HIV infection and certain determinants differs by geographic area [35], the level of poverty and employability are configured as important determinants of HIV prevalence [36] and socioeconomic, demographic and cultural factors evidence changing trends influencing HIV over time [37], among others.

Similarly, there are numerous empirical studies of the association between certain health determinants and the knowledge about HIV/AIDS that an individual or population may possess: among married women, a strong impact of education, access to media, residence, wealth index and employment status on HIV knowledge was detected [38]; among men that have sex with men (MSM), it is evidenced that aspects such as low levels of schooling, non-white ethnicity, belonging to lower economic classes, youth, not having had a screening test and sexual monogamy presented associations with a low level of knowledge about HIV/AIDS [39]; among women of childbearing age, education level was found to be the dominant factor associated with HIV knowledge [40]; as for the adolescent and youth population, although they have a very high risk of HIV transmission during sex, they are poorly informed about HIV and have very negative attitudes towards the virus [41].

Although the particular conclusions given by the previous studies are limited to the context and characteristics of the population under study, the general conclusion suggests that there are associations between the health determinants of individuals and the prevalence and level of knowledge of HIV that should be analyzed to complement the clinical evaluation of the disease [42].

### 2.3. Parametric and Non-Parametric Predictive Modeling of HIV/AIDS

Nowadays, the efforts to develop learning models that could be able to assimilate information from accumulated or high-dimensionality data and predict different factors with greater accuracy and flexibility than conventional multivariate regression models (parametric) have allowed the formulation of other types of predictive models that confer flexibility and unstructured decision making [43], calling them non-parametric classification models.

In the field of public health, one of the underlying concerns of healthcare providers is the expansion of knowledge about HIV status and implications [44]. Therefore, predictive modeling is one of the most effective tools for public policy makers. Hailu [44] posits that health programs cannot provide appropriate HIV/AIDS care, treatment and counseling without knowing who is infected and how much knowledge about their status is possessed. This implies that identifying the best predictive model for these aspects that influence or impact HIV/AIDS is critical.

In that regard, Ahlstrom et al. [23] emphasize that machine learning algorithms, a set of mathematical tools that extract patterns from large data sets to make predictions about the outcome in new or unknown cases, are rapidly growing areas of research that have also made their way into HIV research; they note that these not only improve the discriminatory ability, but can also help identify individuals at higher risk for HIV and with a lower degree of understanding about HIV.

Likewise, Tang et al. [45] found out, in a recently developed technology based on the study of machines in artificial intelligence and databases, the potential for accurate identification of diseases and conditions based on certain important attributes resulting in valuable tools in the medical field: parametric and non-parametric predictive modeling in data mining. They concluded that, in current times, the study of the prevention, diagnosis and treatment of HIV/AIDS has entered a new phase thanks to these trends in predictive modeling discovering potential factors and more efficient treatments for the epidemic [45].

In a comparative perspective between the two types of models, Bao et al. [46] remark that conventional approaches for HIV/STI diagnosis prediction (parametric) are questionable. Therefore, they state that the use of machine learning approaches is a growing trend in HIV/STI research, given that these approaches can incorporate a larger number of covariates in a large data set, handle complex relationships between predictors and the outcome, and achieve a high accuracy [46].

## 3. Methods

### 3.1. Data Source

The data corresponding to the target population for this study are part of the unit of analysis of the 2019 Demographic and Family Health Survey (DHS) [47]. This survey is a database of a complex sample, which is characterized by being two-stage, probabilistic, balanced, stratified and independent, at the departmental level and by the urban and rural area sampling units, to obtain updated information and perform analyses of change, trends and determinants of fertility and mortality, as well as a series of maternal and child health indicators and recent indicators of non-communicable and communicable diseases in Peru [47].

The main aim of the survey is to provide the country with reliable elements and aspects of demographic dynamics, as well as to provide references on the status and factors associated with non-communicable and communicable diseases and for the evaluation and formulation of population and family health programs in the country. The sample size designed to provide representative estimates for the DHS 2019 (annual) is 36,760 homes, with 14,760 homes in the headquarters area (departmental capitals and the 43 districts that make up the Province of Lima), 9340 homes in the rest of the urban area and 12,660 homes in the rural area; the final number of individuals who meet the characteristics of the target population and who have complete information reaches an estimated 10,565 residents in the country that are considered for this survey. Likewise, the sample design specifications should be considered in the analysis process (such as the conglomerate, the stratum and the weighting factor) to obtain an adequate estimation of the indicators [48].

### 3.2. Statistical and Machine Learning Models

#### 3.2.1. Logistic Regression under Complex Survey Data

Suppose that a finite population U={1,2,⋯,N} is divided into h=1,2,⋯,H strata, and each stratum is further divided into $j=1,2,\cdots ,n_{h}$ primary sample units (PSU), each of which is constituted by $i=1,2,\cdots ,n_{hj}$ secondary sample units (SSU), each comprehending $n_{hji}$ elements [49]. Assume also that the observed data consist of $n_{hj}^{\prime }$ SSU chosen from $n_{h}^{\prime }$ PSU in the stratum *h*. The total number of the observation is then given by $n\;=\;\sum_{h=1}^{H}\sum_{j=1}^{n_{h}^{\prime }}\sum_{i=1}^{n_{hj}^{\prime }}n_{hji}$. Each sampling unit has an associated sampling weight given by the inverse of its probability of inclusion in the sample, denoted here by $w_{hijk}=\frac{1}{\pi_{hijk}}$, for the hjik-th unit [49].

Additionally, let $Y_{hjik}$ denote the binary response variable, $x_{hjik}$ denote the covariate matrix and β denote the regression coefficients [49]. Thus, in general, the survey logistic regression model is given by

(1) $$\mathrm{logit}\left\{P\left(Y_{hjik}=1|x_{hjik}\right)\right\}=\ln\left\{\frac{P(Y_{hjik}=1|x_{hjik})}{\left(1-P\left(Y_{hjik}=1|x_{hjik}\right)\right)}\right\}=x_{hjik}^{\prime }\beta .$$

Therefore, under the complex sampling design, the parameter β of the logistic regression model is estimated by the maximum pseudo-likelihood method, also called weighted maximum likelihood, which incorporates the sampling design and the different sampling weights in the estimation of β [49]. The main idea of this method is to define a function that approximates the likelihood function of the sampled finite population with a likelihood function formed by the observed sample and the known samplings weights. In this case the pseudo-log-likelihood function is given by

(2) $$l_{p}\left(\beta \right)=\sum_{h=1}^{H}\sum_{j=1}^{n_{h}^{\prime }}\sum_{i=1}^{n_{hj}^{\prime }}\sum_{k}w_{hjik}\left\{y_{hjik}\times \ln\left[P\left(Y_{hjik}=1|x_{hjik}\right)\right]+\left(1-y_{hjik}\right)\times \ln\left[1-P\left(Y_{hjik}=1|x_{hjik}\right)\right]\right\},$$

where $w_{hijk}$ is the weight of observation hjik. The maximum pseudo-likelihood estimator of β is obtained by deriving the pseudo-log-likelihood function such that β equals zero, $\left(\beta \right)=\frac{d}{d\beta }l_{p}\left(\beta \right)=0$ [49].

In addition, under complex sampling designs, there is not a direct form to calculate the variance estimators. Thus, to obtain the variance estimators using maximum pseudo-likelihood, the Taylor linearization method (also called the delta method) was used (as implemented in the R *survey* package) [49]. The hypothesis tests for the significance of the regression coefficients and the test for the goodness of model fit also need to be modified to incorporate the sampling design and the different weights of the observations. The evaluation of the contribution of the covariates is now made with the adjusted Wald test. Furthermore, in order to obtain valid inferences using this type of design, the Pearson’s test statistic was introduced, such as the Rao–Scott adjustments [49].

#### 3.2.2. Logistic Regression

The logistic regression model directly estimates the probability of occurrence of a dichotomous dependent variable $Y_{i}\left(Y_{i}=1\right)$ given the values of the independent variables $X_{i}$ applying the maximum likelihood estimation procedure to estimate the Y parameter of interest [50]. The relationship between the variables Y and X would be posed as follows as a logistic distribution function:

(3) $$Pr\left(Y=1/X\right)=\frac{1}{1+e^{-(\beta_{0}+\beta_{1}X_{1i}+\beta_{2}X_{2i}+\cdots +\beta_{n}X_{ni})}}=\frac{1}{1+e^{-Y}},$$

where Pr(Y) is the probability of occurrence of Y and e is the base of the natural logarithm. $\beta_{0}$ represents the lateral displacements of the logistic function, $\beta_{i}$ are the coefficients that weight the independent variables and on which the dispersion of the function depends and $X_{i}$ are the independent variables [51].

#### 3.2.3. Artificial Neural Networks

Artificial neural networks (ANN) are composed of a set of highly interconnected simple processors called nodes or neurons, which are organized in layers (input, hidden and output) that allow the processing of information at a given connection or synapse with another neuron [51].

Thus, the total input signal $y_{i}$ to each of the *q* neurons of the intermediate layer will be calculated by summing the input values weighted by their corresponding weights [51]. Subsequently, a nonlinear function called the activation function is applied to this input, thus obtaining the output value of each intermediate node, which, in turn, will be transmitted to the output neuron through the corresponding weighted connection [51]. Where $F(y_{i})$ is the output of each intermediate node under the activation function *F*, *y* is the network output and $\beta_{i}$ are the connections to the output layer,

(4) $$y=\beta_{i}F\left(y_{i}\right).$$

#### 3.2.4. Random Forest

According to Cutler et al. [52], the random forest model is a tree-based set of binary recursive partitions in which each tree depends on a collection of random variables. For a *p*-dimensional random vector $X={\left(X_{1},\cdots ,X_{p}\right)}^{T}$ representing the real-valued input or predictor variables and a random variable *Y* representing the real-valued response, we assume a joint unknown distribution $P_{XY}\left(X,Y\right)$ [52]. The objective is to find a prediction function f(X) to predict *Y*. The prediction function is determined by a loss function L(Y,f(X)) and defined to minimize the expected value of the loss:

(5) $$P_{XY}\left(L\left(Y,f\left(X\right)\right)\right),$$

where the subscripts denote the expected value with respect to the joint distribution of *X* and *Y* and L(Y,f(X)) is a measure of how close f(X) is to *Y* [52].

#### 3.2.5. Decision Tree

Decision trees are a non-parametric binary classification technique that combines the characteristics of the classic univariate model and those of multivariate systems [51]. The authors define that the process consists of successively dividing the original sample into subsamples using univariate rules that will search for the independent variable that best discriminates the division [51]. In order to find the best division rule, the algorithm will study each of the explanatory variables, analyzing cut-off points in order to be able to choose the one that provides the greatest homogeneity to the new subgroups under the premise of minimizing the impurity of the node. The process ends when it is impossible to make a new division that improves the existing homogeneity [51].

#### 3.2.6. k-Nearest Neighbors Algorithm

According to Zapata et al. [53], the k-nearest neighbors algorithm is based on the properties of an input datum *x* that are similar to those of the data in its neighborhood, so it belongs to the same class as the most frequent class of its *k* nearest neighbors. The general algorithm of the *k*-nearest neighbors method assumes that all observations correspond to points in a *p*-dimensional space $R_{p}$, which have a class C set [53]. The data are of the form presented below:

(6) $$\left(x_{i},c_{i}\right)=\left(c_{i1},c_{i2},\cdots ,x_{ip},c_{i}\right).$$

### 3.3. Study Methodology

**Figure 1:**
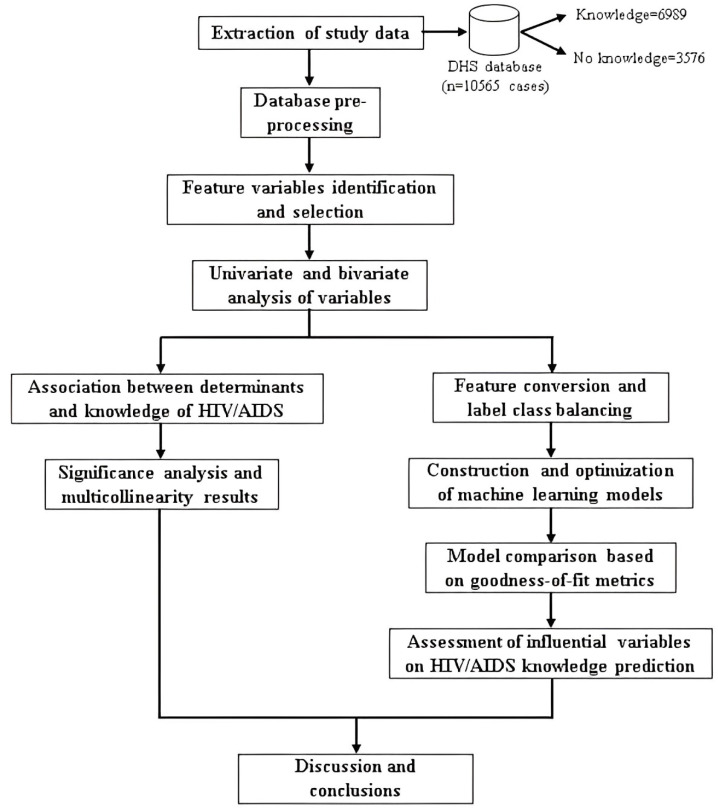
Study methodology diagram for the study of HIV/AIDS knowledge among the adolescent and young adult population in Peru.

The methodology proposed for the fulfillment of the study objectives, as shown in Figure 1, consists of: (i) collection of the study data; (ii) pre-processing of the database; (iii) variable identification and selection; (iv) statistical association between determinants and HIV/AIDS knowledge; (v) construction and optimization of machine learning models for predicting HIV/AIDS knowledge and (vi) discussion and conclusions of the research.

#### 3.3.1. Database Preprocessing

Considering the unification process to obtain the study database, the essential study indicators are mainly present in the CSALUD01 health status and conditions module of the DHS. The module houses data from individuals aged 15 years and older on various conditions, risk factors, perception and knowledge of non-communicable and communicable diseases and mental health, among others. In addition to this module, additional modules that make up the DHS such as Household, People and Women are necessary to unify, complete and filter the health database in order to be able to analyze the survey correctly and directly [48].

On the other hand, the dataset had several unuseful features, such as the identifiers created to merge the previous modules and individual characteristics that were not part of the literature review conducted prior to the study. Such features were excluded, and 15 features were included in the final model. Like many census data, the DHS data often contain variables with missing observations. All variables had some level of missingness, which ranged from 5% to 20% of the observations in certain cases. The records containing at least one missing value were eliminated from the final dataset.

#### 3.3.2. Variable Identification

The dependent variable of the study is the knowledge about HIV/AIDS that adolescents and young adults in the country have, described in Table 1. An individual has an adequate knowledge about HIV/AIDS if he/she gives correct answers to questions about the transmission and prevention of HIV/AIDS asked in the health module of the survey.

Furthermore, the determinants included in the model can be widely classified into socio-demographic characteristics, health determinants and economics determinants, defined in Table 1.

The socio-demographic characteristics encompass the gender of the respondent, geographical location of the respondent (urban or rural), region of residence (in the Peruvian case, it comprises the capital of the country, coast, highlands and jungle), age of the respondent, highest educational level of the interviewee, ethnic self-perception of the respondent, marital status of the interviewee, the gender of the household head, nationality of the interviewee and the respondent’s first language. The economic determinants include the wealth quintile of the interviewee and access to mass media (which comprises access to radio, television or internet). Finally, the health determinants comprise whether the interviewee received any information regarding HIV/AIDS in the past and whether the respondent had a previous HIV screening test.

#### 3.3.3. Statistical Analysis of the Determinants Associated with Knowledge of HIV/AIDS

A univariate and bivariate analysis will be performed to obtain a perspective and knowledge about the data. In the case of univariate analysis, frequency distribution tables will be used to identify the number and percentages of individuals in a sample that meet certain categories of the regressors studied. In the case of bivariate analysis, associations between variables will be evaluated using the Chi-square statistic; if the contigency table, which compares two categorical variables, presents a value of less than 5 in one of its cells, Fisher’s statistic will be used to determine the relationship between the factors in question.

#### 3.3.4. Association between Structural Determinants and Knowledge about HIV/AIDS

In order to determine how socio-demographic, economic and health variables affect the level of knowledge of adolescents and young adults in Peru, a quasi-binomial logistic regression under complex survey data will be fitted to the dataset extracted from the DHS survey, considering the individual characteristics defined in Section 3.3.2 as the dependent variable and independent factors.

The assumptions for the application of this method are the following: (i) Regarding the application of logistic regression to survey data, the standard logistic regression model is inappropriate when the data refer to samples from complex sampling designs [49]; thus, a logistic regression for complex survey data is a suitable option. (ii) For the use of a quasi-binomial family in the logistic regression, in statistical terms, when the value of the dispersion parameter φ is greater than unity in binary response scenarios such as in the context of samples obtained with complex design methods (non-integer counts produced by the use of differential sampling weights), it indicates that the model is over-dispersed and that the model parameters may be underestimated [54]; hence, a logistic regression based on a quasi-binomial family becomes the ideal choice to deal with this particular situation by modelling over-dispersion. (iii) In computational terms, a logistic regression with a binomial family assumes that the weight or contribution of each observation in the weighted probability within the model is an integer; however, when the weighting factors result in non-integers (as occurs in surveys with a complex design such as the DHS survey of this study), the model processing fails to fit a result to the observations [54]; therefore, the quasi-binomial family is the family variant that accepts these non-integer contributions and reaches a model fit in this situation.

The results of the regression coefficients, the standard errors, the t-test statistic based on the adjusted Wald test, the significance level associated with the variables, the adjusted odds ratios and the diagnosis of multicollinearity (using the generalized variance inflation factor (GVIF)) will be reported.

#### 3.3.5. Preprocessing of the Data Set for the Application of Computational Models

First, the conversion of categorical independent variables to binary values under one-hot encoding is required to convert categorical variables into binary representations. Such a representation was chosen to facilitate the future reduction of variables in models that require it and to minimize the impact on the model structure (referring to the complexity that an algorithm can assume for the given variables).

On the other hand, in order to provide a good generalization and adjustment of the proposed algorithms for the prediction of an individual’s HIV/AIDS knowledge level, the original data set, composed of 10,565 observations, is divided into two new sub-samples: a training set of 90% of the data (9509 records) and a test set grouping the remaining 10% (1056 records). However, considering that the training set generated suffers from an imbalance problem in the response variable (since the class of interest “adequate level of knowledge” only represents 34% of the total observations), the sampling method chosen in order to balance the classes of the dependent variable is the SMOTE (Synthetic Minority Oversampling Technique) [55]. Under the sampling treatment, the minority class (adequate knowledge) would be oversampled from 3218 individuals to 6436 individuals and the majority class (inadequate knowledge) from 6291 to 6436 individuals: the new training set would be composed of 12,872 records.

#### 3.3.6. Construction and Comparison of Computational Models

The classification models selected from the existing set of traditional machine learning techniques are logistic regression (LR), artificial neural networks (ANN), decision trees (DT), k-NN algorithm (k-NN) and random forest (RF). For all five models, we pose the research objective as a two-class problem, such that each value in the label is binarized.

In order to determine the ideal structure and composition for the exposed algorithms, a 10-block model-fitting and cross-validation process was carried out to analyze the difference in different fitting iterations. For each algorithm that was evaluated, the parameters taken into account and their definitions are listed in Table 2. Nevertheless, in the case of the logistic regression, no hyper-parameter was included since it only assumes a link function between the response variable and the covariates, lacking parameters that modify the predictive capacity of the technique and affect its performance.

Since various metrics capture different characteristics of a classifier, depending on the properties of the data, the choice of metrics influences how the performance of classification algorithms is measured and compared [56]. In this case, the common or traditional metrics to be used to compare techniques are accuracy, sensitivity (also called recall or true positive rate—TPR), specificity (or true negative rate—TNR), false negative rate (FNR), false positive rate (FPR), Cohen’s kappa, F1 score and AUC (area under the curve). The formulas for calculating the performance metrics are as follows:

(7) Positivecase(PC)=AdequateHIV/AIDSknowledge

(8) Negativecase(NC)=InadequateHIV/AIDSknowledge

(9) Truepositive(TP)=Numberofpositivecasespredictedaspositive

(10) Truenegative(TN)=Numberofnegativecasespredictedasnegative

(11) Falsepositive(FP)=Numberofnegativecasespredictedaspositive

(12) Falsenegative(FN)=Numberofpositivecasespredictedasnegative

(13) Accuracy=(TP+TN)÷(TP+TN+FP+FN)

(14) Recall/TPR/Sensitivity(SENS)=TP÷(TP+FN)

(15) TNR/Specificity(SPEC)=TN÷(TN+FP)

(16) Falsepositiverate(FPR)=FP÷(FP+TN)

(17) Falsenegativerate(FNR)=FN÷(FN+TP)

(18) $$\mathrm{Observed}\;\mathrm{precision}\;(P_{o})\;=\;(\mathrm{TP}\;+\;\mathrm{TN})\;÷\;(\mathrm{TP}\;+\;\mathrm{TN}\;+\;\mathrm{FP}\;+\;\mathrm{FN})\;\;$$

(19) $$\mathrm{Expected}\;\mathrm{precision}\;(P_{e})\;=\;\frac{(\mathrm{TP}\;+\;\mathrm{FP})(\mathrm{TP}\;+\;\mathrm{FN})\;+\;(\mathrm{FN}\;+\;\mathrm{TN})(\mathrm{FP}\;+\;\mathrm{TN})}{{(\mathrm{TP}\;+\;\mathrm{TN}\;+\;\mathrm{FP}\;+\;\mathrm{FN})}^{2}}\;$$

(20) $$\mathrm{Cohen}’s\;\mathrm{kappa}\;=\;(P_{o}\;-\;P_{e})\;÷\;(1\;-\;P_{e})\;\;$$

(21) F1score=(2×TP)÷(2×TP+FP+FN)

(22) AUC=(1−SPEC)×SNES÷2+(SENS+1)×SPEC÷2

Once a set of influential characteristics has been identified, the next step is to summarize the functional relationship between each characteristic, or subset thereof, and the outcome of interest [57]. For such work, the local correlation value will be used to specify the role of each attribute in the prediction of a response variable and thus generate a relevance ranking of the factors considered, with positive values indicating that the attribute supports the correct predictions and negative values suggesting that the feature contradicts them [58]. Those levels that exceed this value (> 0.01) will be considered as empirically positive [58], thus specifying that these support positive predictions in the observations related to the level of adequate knowledge about HIV/AIDS in adolescents and young adults in Peru.

### 3.4. Analytical Tools

The statistical analyses were carried out with the statistical software RStudio [54] using the *survey* package [59]. On the other hand, the classification and prediction models of the knowledge of the individuals were carried out using the analysis and data mining software RapidMiner [58].

**Figure 2:**
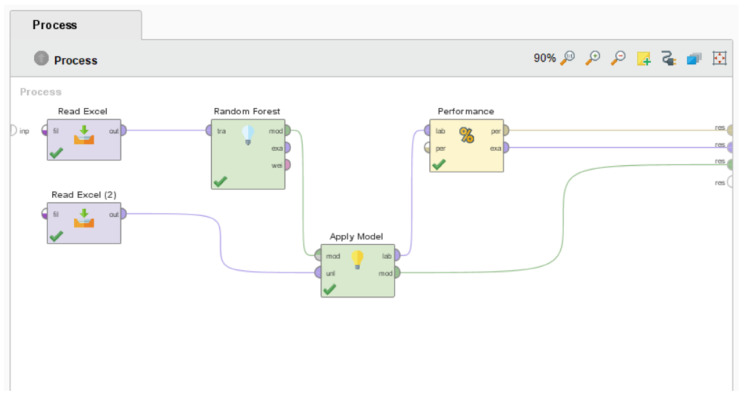
Design view of the construction of a working process for a standard computational model in RapidMiner, according to [58].

Considering the latter software, RapidMiner is a free, ready-made and open-source software tool for data and text mining [58]. It is an analytics platform with a visual workflow design and full automation written in Java and contains more than 500 pre-defined operators with different approaches to accelerate the creation, delivery and maintenance of high-value predictive analytics [58]. Its visual programming system, known as Drag & Drop, requires a lower learning curve achieving higher productivity in less time: it allows one to swiftly construct data-mining applications without coding (or the application of a language) or programming skills; by dragging process blocks onto a canvas and linking them all together, it is more feasible to prepare data for the final output and visualize it [58], as shown in Figure 2

In that sense, the choice to use RapidMiner as the software for the development of the computational models is based on the fact that it offers fully customizable processes that go far beyond simple application scenarios and flexibility through its graphical user interface, with no programming involved at the bottom level, making our research easier and more accessible [58].

## 4. Results

### 4.1. Statistical Analysis of Factors Associated with Knowledge about HIV/AIDS

According to Section 3.3.3, Table 3 shows the results of the level of knowledge about HIV/AIDS of the interviewed population aged 15–29 years according to the characteristics of the sample collected in the DHS database.

From a sample of 10,565 individuals, there is a significant contrast in the distribution of the level of knowledge about the virus and the disease among the target population, estimating that only 3576 (33.80%) respondents had an adequate level of knowledge and the rest of the people (6989 (66.20%)) had incorrect notions or perceptions about the epidemic. Considering socio-demographic, economic and health factors, differences can be seen in terms of knowledge about the epidemic, both as a result of percentage sample estimates (which ignore the complex design of data collection) and weighted estimates (which include weights and the structure of the sampling frame).

On the other hand, taking into account the results of the independence tests of Pearson’s $\chi^{2}$ statistic, it was established that the variables Marital status ($\chi^{2}$ = 3.087, *p*-value = 0.499) and Nationality ($\chi^{2}$ = 4.047, *p*-value = 0.275) did not show a significant association or statistical relationship with the response variable since both *p*-values shown above exceed the confidence levels considered for the study, so they were dismissed or discarded from the subsequent logistic regression model. The remaining independent variables showed a significant association with the dependent variable, considering the obtained *p*-values.

### 4.2. Association between Structural Determinants and Knowledge about HIV/AIDS in Peru

The results of the quasi-binomial logistic regression employed to measure the relationship between the knowledge about HIV/AIDS and the independent variables, described above, are analyzed with a significance level (*p*-value) of 10%, 5% and 1% and reported in Table 4.

Gender is a significant predictor of the knowledge among adolescents and young adults in the country (*p* < 0.01). Being of the male gender is negatively correlated with the probability of having an adequate and correct understanding of HIV/AIDS (β = −0.334).

The regression coefficients associated with the economic-level categories show a positive association with the probability of possessing knowledge and these variables are significant (*p* < 0.01).

There is a propensity in those individuals living in the Sierra region of Peru not to possess a correct level of knowledge about the interaction with HIV/AIDS and its forms of transmission (they are negatively correlated with the response variable considering that β = −0.178).

Those within the age range of 25 to 29 years have a significant positive correlation (*p* < 0.01) with the dependent variable (β = 0.285).

The results in terms of the highest educational level attained significantly demonstrate that individuals who completed high school (β = 1.911, *p* < 0.05) and higher education (β = 2.198, *p* < 0.01) are more likely to have an adequate knowledge than those individuals without any accredited education (or illiterate).

Adolescents and young adults who self-identify as Afro-Peruvian (β = −0.328, *p* < 0.05) or other ethnicity (β = −0.387, *p* < 0.05) are less likely to have an appropriate knowledge than those who self-identify as indigenous or of native origin in the country.

Having heard some type of information regarding HIV/AIDS has a significant positive impact on determining the knowledge of these that an adolescent or young adult may have (β = −0.635, *p* < 0.01).

Adolescents’ and young adults’ performance of an HIV/AIDS screening test is positively linked to the individual’s adequate knowledge about the epidemic (β = 0.174, *p* < 0.05).

Finally, having Spanish as a primary language shows a positive and significant correlation (β = 0.233, *p* = 0.052) with the dependent variable. However, speaking a foreign language as a primary language is negatively associated (β = −1.915, *p* = 0.082).

In contrast, area of residence, access to mass media and gender of the head of household are not correlated with the knowledge about HIV/AIDS (*p* > 0.10).

Considering the variances of the estimated regression coefficients, it can be established that there is no multicollinearity problem in the model, since the GVIF values are less than 5 [60].

### 4.3. Construction and Optimization of Computational Models

**Figure 3:**
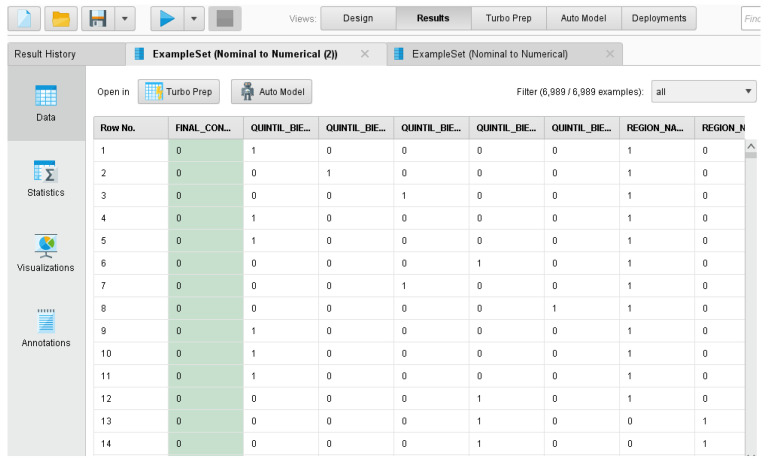
Results of the one-hot encoding conversion of variables in this study through the use of the software RapidMiner and the Nominal to Numerical Operator.

Considering the representation of categorical variables chosen on Section 3.3.5 to minimize the impact on the structure of the proposed models, Figure 3 presents the data-transformation process carried out in the study using the software RapidMiner.

On the other hand, based on the selection of available hyper-parameters to be optimized and the cross-validation procedure presented above, the available test alternatives for each model and the selected value that optimizes each of them are given in Table 5.

These models (with the specifications presented above) were generalized to the test set to determine comparatively which one best allows classification of the knowledge about HIV/AIDS of adolescents and young adults in the national territory.

**Figure 4:**
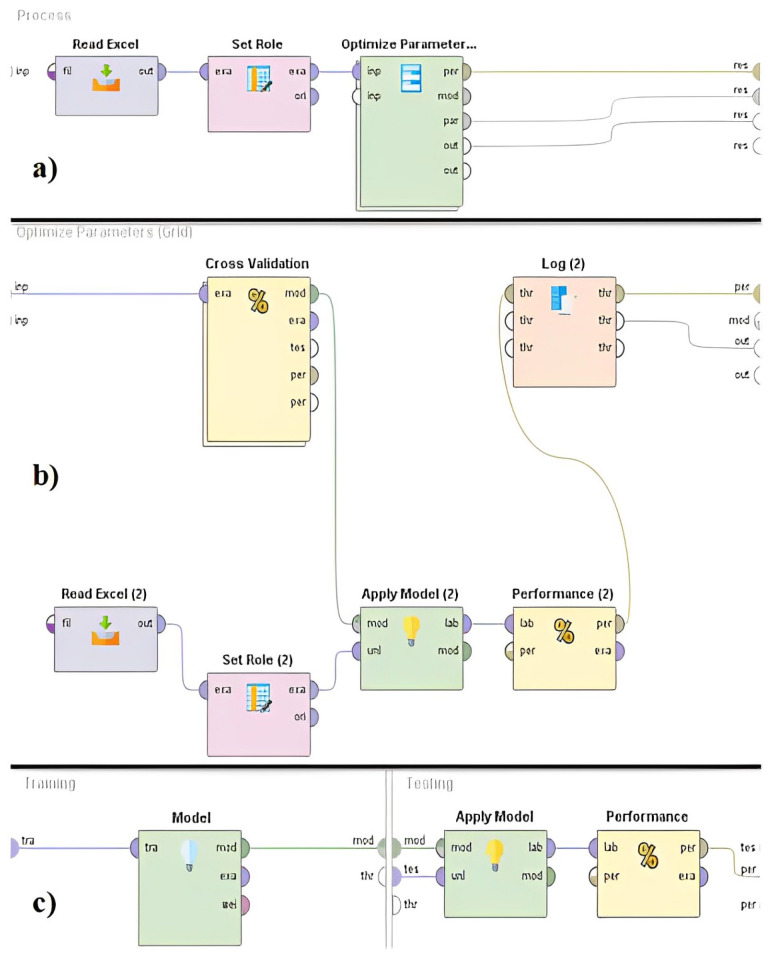
Hyper-parameter tuning architecture for parametric and non-parametric models in the study. (**a**) Refers to the parameter tuning process with the Optimize Parameters (Grid) parameter. (**b**) Refers to the cross-validation process in model evaluation with the Cross Validation operator. (**c**) Shows the training and validation process within cross validation and generation of performance metrics.

In that perspective, subsequent to the constraint of the hyper-parameters to be used in each algorithm in Table 2, a process of model fitting using grid search and cross validation was carried out. The architecture of the workflow performed in RapidMiner is shown in Figure 4.

### 4.4. Comparison of Classifiers for the Prediction of Knowledge about HIV/AIDS

**Figure 5:**
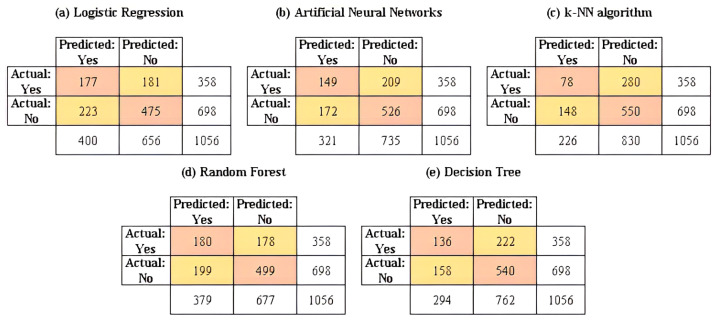
Confusion matrices for the machine learning models developed for the prediction of HIV/AIDS knowledge in the test set.

Once the training process is completed, the validation of the predictive performance of the machine learning models is performed by generalizing them to the test data. The results of the confusion matrices for the machine learning models considered in this study are reported in Figure 5. In addition, the results of the computational models built in the training stage and applied to the test set are reported in Table 6.

Regarding the goodness-of-fit indicators considered in this study, it can be established that the random forest method presents the best scores in each indicator (except for the AUC value, FPR and specificity). This algorithm shows the greatest predictive performance in contrast to the other parametric and non-parametric models included in this study.

**Figure 6:**
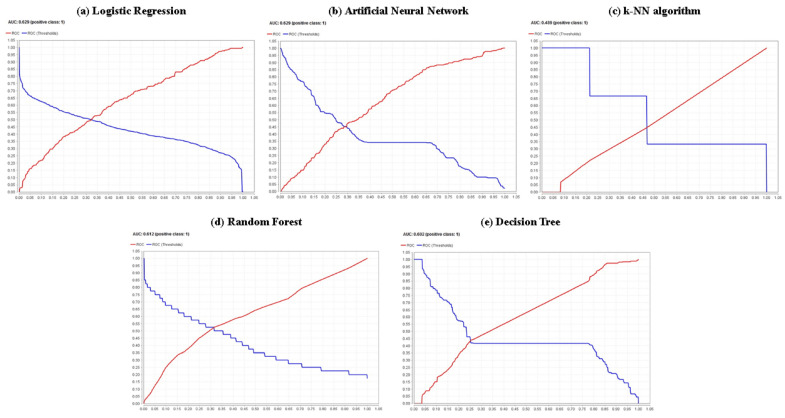
ROC curves and AUC values associated with the parametric and non-parametric classification models for the test set.

The highest identification of the number of true positives (TP) was using the random forest method (180 cases); the highest identification of the number of false negatives (FN) also occurred in the random forest method (178 cases). Regarding the false negative rate (FNR), the lower value (49.72%) was achieved by the random forest model; considering the accuracy, the highest value (64.30%) was reached by the random forest model. Regarding the sensitivity, it can be pointed out that the random forest model has the highest percentage or proportion of correct positive predictions among the total positive predictions among all the models used (50.28%); Analyzing the specificity, it is noted that the k-NN algorithm is the model with the best performance in terms of this goodness-of-fit metric, with a value that amounts to 78.80%. From another point of view, examining metrics such as Cohen’s kappa and the F1 score, it can be shown that the random forest model is the one that presents the best performance in both indicators, with values of 0.215 and 48.85%, respectively. In the same sense, a visualization of the receiver operating characteristics (ROC) curve is shown in Figure 6; considering the AUC, the curves of the binomial regression and artificial neural network models show the highest AUC value (62.90% for both cases), indicating they are the best models for classifying the knowledge and lack of knowledge of HIV/AIDS at the target population, among the models. However, it is necessary to remark that the random forest model offers the second-best value for said indicator with 61.20%, with a minimal difference of 1.70% and, in the same line, for high levels of specificity, this same model obtains moderate levels of sensitivity. Only in the cases of metrics such as the number of true negatives (TN), the number of false positives (FP) and the false positive rate (FPR), the k-nearest neighbors algorithm offered the best fit with 550, 148 and 21.20% values for these indicators, respectively.

### 4.5. Identification of Variables That Influence the Prediction of HIV/AIDS Knowledge

**Figure 7:**
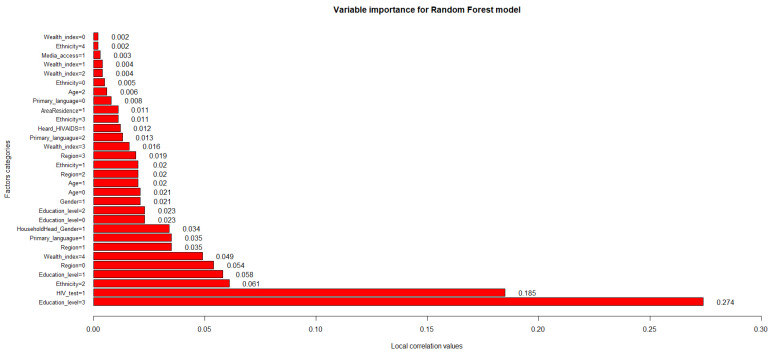
Local correlation values of the independent factor categories related to the prediction of the knowledge about HIV/AIDS in the target population in the random forest model.

Regarding the variables’ influence on the performance of the best predictive model obtained, Figure 7 shows the importance values of the factor categories used in this study.

The random forest, being the best model evaluated, allowed us to establish the characteristics or factors that have the greatest influence over the predictive capacity (the performance of the classification predictions) and the performance in estimating the knowledge about HIV/AIDS in adolescents and young adults in Peru: urban area of residence (AreaResidence = 1, correlation value = 0.011); Afro-Peruvian ethnic self-perception (Ethnicity = 3, correlation value = 0.011), Caucasian ethnic self-perception (Ethnicity = 1, correlation value = 0.02) and mixed ethnic self-perception (Ethnicity = 2, correlation value = 0.061); having previously heard information about HIV/AIDS (Heard_HIVAIDS = 1, correlation value = 0.0.012), having Spanish (Primary_language = 1, correlation value = 0.035) and a foreign language as a primary tongue (Primary_language = 2, correlation value = 0.013); belonging to the second highest (Wealth_index = 3, correlation value = 0.016) and highest wealth indexes (Wealth_index = 4, correlation value = 0.049); living in the capital (Region = 0, correlation value = 0.054) or in inland regions of the country (Region = 1, Region = 2 and Region = 3 with correlation values of 0.035, 0.02 and 0.019, respectively); age ranges of 15–20 (Age = 0, correlation value = 0.021) and 21–24 (Age = 1, correlation value = 0.02); being of the male gender (Gender = 1, correlation value = 0.021); all established educational levels (Education_level = 0, Education_level = 1, Education_level = 2 and Education_level = 3 with correlation values of 0.023, 0.058, 0.023 and 0.274, respectively); head of household of the male gender (HouseholdHead_Gender = 1, correlation value = 0.034); and the performance of prior HIV/AIDS screening tests (HIV_test = 1, correlation value = 0.185).

Thus, it can be specified that the obtained values of local correlations between 0.01 and 30.00% are the most feasible and beneficial to use within the random forest since they allow estimation of the response variable by positively influencing the model’s predictive performance, emphasizing that having a higher education level is the most relevant factor (highest correlation value = 0.274) among the considered variables.

## 5. Discussion

This study aimed to describe the characteristics of adolescents and young adults in Peru according to their knowledge about HIV/AIDS, identify structural determinants of health that possess an influence on this knowledge and build a computational model to estimate the knowledge of HIV/AIDS in the target population through a comparison of parametric and non-parametric techniques.

As an overall result, the association between certain socio-demographic, economic and health factors and the level of knowledge about the forms of prevention and rejection of misconceptions about HIV/AIDS transmission in Peru was established. On the other hand, the predictive capacity of the level of knowledge about the epidemic was estimated as between 59.47% and 64.30% for the models considered, with the random forest model (64.30%) being the one that showed the best performance. In addition, the study showed that this algorithm allows identification of the following main variables that have an influence on the prediction of HIV/AIDS knowledge: gender of the respondent, area of residence of the interviewee, wealth index of the respondent, region of residence, interviewee’s age, highest educational level attained by the respondent, ethnic self-perception of the interviewee, having heard about HIV/AIDS in the past, the performance of an HIV/AIDS screening test by the respondent, mass media access by the interviewee, the gender of the household head and the primary language of the interviewee.

First, this research showed that the level of knowledge associated with HIV/AIDS among Peruvian adolescents and young adults aged 15–29 years can be considered low, given that only 3576 individuals out of the entire study sample reported an appropriate knowledge of the epidemic, which translates into 33.80% of the cohort having a correct understanding of the risks posed by the virus and the forms of prevention and sexual care that can be used to avoid infection and respond proactively to HIV and people living with the disease. Such categorization of low level of knowledge coincides with other research conducted elsewhere, which obtained similar findings in terms of the percentage of the sample that effectively reported a correct understanding of the dimensions of the epidemic: Shokoohi et al. [61] (37.30%), Dadi et al. [62] (30.31%) and De Wet et al. [63] (10.00%). Furthermore, in a study published in Peru, Becerra et al. [64] reported a correct understanding of 33.3%.

Second, the current study identified the socio-demographic, economic and health variables that significantly predict (under a significance level of *p* < 0.10) the probability of a young adult having an adequate knowledge about HIV/AIDS in Peru, which are shown in Table 4.

It was established that males are less likely to have an adequate perception of HIV/AIDS. Research conducted in 45 countries around the world, through survey analysis, shows that there is a steady increase in knowledge of HIV prevention and concepts among young women, surpassing that recorded, over time, by men [65]. In the same vein, a study in Brazil that explores the factors associated with the knowledge, attitudes and practices of young Brazilians about HIV, STIs and viral hepatitis points out that young Brazilian men aged 18–29 years are more likely to adopt risky sexual behaviors than young women as they have a lower level of health knowledge [66].

The results show that the higher the level of income or wealth, the higher the probability of having an adequate level of knowledge about the epidemic increases progressively. A study conducted in Nigeria based on economic inequality as a predictor of HIV-related knowledge concurs with the findings obtained, establishing that the probabilities of low HIV-related knowledge increase significantly in each wealth category as wealth decreased [67]. In the same perspective, a cross-sectional analysis of youth aged 15–24 years in Nigeria shows that youth from middle and wealthy households, respectively, had significantly higher HIV-related knowledge than youth from poor households [68].

It is noted that those individuals coming from the natural region of the Sierra are less likely to possess correct knowledge about HIV/AIDS. Research conducted in Pakistan shows that the region of residence recorded a significant relationship with HIV/AIDS knowledge in young respondents, in which it is pointed out that the level of discernment was higher in the capital city than in the provinces of the country [69].

It is recognized that those respondents with education levels of secondary school and above are more likely to be aware of the transmission and risks associated with the epidemic. Findings from a study considering adult individuals aged 15 years and older in Pakistan and Afghanistan specify that, in both countries, education is a fundamental factor in HIV knowledge, establishing that people with higher levels of education were more likely to have accurate and comprehensive knowledge about HIV/AIDS, emphasizing that understanding about various aspects related to the epidemic increases when the educational level of the person increases progressively [70].

It is established that those individuals who were tested for the virus prior to the survey are more likely to have adequate perceptions of HIV/AIDS progress and prevention. An analysis conducted in Iran, based on the assessment of HIV-related attitudes, practices and knowledge among young men and women, indicates that, given the limitations of Iranian youth in terms of sexual health and risk practices linked to STIs and HIV/AIDS, the low prevalence rates of testing for the virus express low levels of knowledge of the epidemic and erroneous attitudes about it, which calls for specific interventions to increase the acceptability of testing [61].

Those adolescents and young adults who have inadvertently come into contact with some information about HIV/AIDS previously are more likely to have adequate knowledge about the epidemic. A study developed in South Sudan on the knowledge, attitudes and practices related to HIV/AIDS among adolescents in the country determines that, in general, the vast majority of young students who have good knowledge about HIV/AIDS have heard about HIV/AIDS through different avenues (such as school, media or their parents); thus, promoting the increase in the level of understanding of the epidemic with increased sex education and contact with information regarding the virus is recommended [71].

Those who speak Spanish or who have Spanish as their primary language have a higher level of understanding than individuals who speak dialects native to Peru; conversely, those who have a foreign language as their native language are less likely to have an appropriate understanding of the epidemic. A study conducted in Indian adolescents on their knowledge and attitudes about HIV/AIDS and STIs indicates that the language taught and spoken at home had an important relationship with knowledge of the epidemic, emphasizing that those who spoke English as a foreign language were less likely to have correct perceptions about HIV/AIDS than those who had a Hindi dialect as their native language [72]. Likewise, Rachlis [73], in relation to individuals who had dialects and/or native languages as their primary language in Canada, indicates that there are barriers related to native languages that result in a low level of knowledge about HIV/AIDS in these people because the absence of a common language between people who are part of these isolated communities and the bulk of the population with a consolidated and massified language generates an inability to communicate clearly and directly.

It is noted that those individuals who self-identify as Afro-Peruvian or who do not self-identify through one of the categories that the state offered in the questionnaire are less likely to have an adequate understanding of HIV/AIDS. Research conducted in London based on young people from ethnic minorities indicates that they are far from being a homogeneous group and that they are related to different ethnic differences in sexual health knowledge; namely, the lowest levels of sexual health knowledge about HIV/AIDS that represent a major concern for the British authorities are recorded in the black African youth population, white minorities of unidentified ethnicity and individuals from Asian areas with relevant knowledge gaps [74].

It is determined that belonging to the age range of 25 to 29 years represents a higher probability of having an adequate perception about HIV/AIDS than belonging to the reference range. Shokoohi et al. [61], in their study of the practices, attitudes and knowledge associated with HIV/AIDS in individuals of the youth segment in Iran, detailed that participants aged 25–29 years possessed much more knowledge about ways of transmission, prevention and perception of HIV compared to other age groups, reflecting that knowledge and positive attitudes about HIV increased with age, which could be attributed to older people’s greater interest in seeking information about sexual health or greater exposure to sexual health education.

Third, this research unveiled that, subsequent to a literature review in the health field, it is noticeable that little work has been conducted on the assessment and intervention research of an understanding of the HIV/AIDS epidemic employing machine learning methods or, in particular, an approach based on a random forest model. The accuracy linked to the prediction of classifications of the level of adequate knowledge about HIV/AIDS is between moderate limits previously reported in the field of healthcare and medical research [75,76,77,78], with values between 59.47% and 64.30% generated in the validation set. The final results of the computational experiments indicated that the random forest had the best predictive performance among all the proposed algorithms, with the highest goodness-of-fit metrics, such as accuracy (64.30%), sensitivity (50.28%), Cohen’s kappa (0.215) and F1 score (48.85%). This could be due to the remarkable properties and benefits offered by the random forest as a machine learning model. First, this technique has the advantage of obtaining better levels of predictive performance and robustness by obviating the need for a cross-validation process because they produce an unbiased estimate of the test set error internally by constructing many bootstrap samples from the original data [79]. Second, this algorithm has the advantage of preventing over-fitting by reducing inter-tree dependence, which makes majority voting an effective strategy in forest construction [79]. Third, random forest can automatically handle continuous, nominal, ordinal, and missing independent variables, capturing nonlinear effects and interaction terms with an ability to adaptively use a large number or dimensionality of covariates, even if most are correlated [80]. In the same perspective, the random forest technique allows quantitatively assessing the contributions of predictor variables to the response variable for the selection of relevant co-factors within a model importance analysis covering the impact of each predictor variable individually, as well as in multivariate interactions with other predictor variables [81]. Finally, the random forest algorithm reduces the risk of overall bias in its development since there are several trees and each tree is trained on a subset of the data [82] and it is relatively stable in the face of etiological variability and a reasonably low amount of missed data [15].

Fourth, by examining whether parametric and non-parametric algorithms could learn from existing national data and help predict the level of HIV/AIDS knowledge, our research presented an approach that allowed us to establish the most predictive features for the level of knowledge by considering the complex nature of various predictors of HIV/AIDS understanding to provide an intuitive understanding of the key features. The random forest, being the best model evaluated, allowed us to establish the characteristics or factors that have the greatest influence on the predictive ability (the performance of the classification predictors) and the performance in estimating the level of knowledge about HIV/AIDS in adolescents and young adults in Peru. Analyzing the most relevant characteristics (the ten categories with the highest correlation value), the following can be established: In the case of economic status or wealth distribution, Pellowski et al. [83] suggest that a person’s economic status may affect the likelihood of having adequate knowledge about HIV/AIDS by affecting their quality of life. In terms of primary language, HIV prevention and knowledge is a major priority in communities that are still recovering from the impacts of the existence of a language barrier that inhibits the proper access to the healthcare system [84]. In relation to the educational level, education protects against HIV infection through information and knowledge that can affect long-term behavior change [85]. Considering the region of residence, the effects of residential segregation and regional disparities combine to negatively impact the knowledge about HIV/AIDS that individuals may have [86]. In reference to ethnicity, a challenge that remains in the delivery of HIV/AIDS prevention and knowledge-building interventions is the ability to incorporate measures to address the unique needs of diverse members of social communities [87]. In terms of HIV/AIDS screening, this health tool can help prevent HIV infection through counseling to discourage high-risk behavior and support protective behaviors and proper sex education [88].

## 6. Conclusions

The results obtained in this study suggest the usefulness of the associations found between structural determinants and the suitability of machine learning models for characterizing and predicting knowledge of HIV/AIDS in the target population and are relevant for policy makers and health-promotion decision makers working at the Peruvian government to provide a starting point for reinforcing, justifying, directing and supporting the planning and execution of improvements and new measures in the efforts to counteract the HIV/AIDS epidemic in the country. It provides grounds for the identification of potential focuses of attention of current and new health policies based on those determinants that exhibit an association with the knowledge that individuals may have about HIV/AIDS, for the evaluation of computational models for the prediction of this knowledge in a prospective way, and for the detection of relevant factors that influence the predictive performance of the models, thus representing the first initiative for the evaluation of the role of the Peruvian government in relation to this public health problem and for possible ways to optimize and encourage an efficient control and reduction in this problem in the future. Future research should consider supplementary techniques and/or methods to those previously presented to analyze the epidemiological situation of HIV/AIDS in Peru and to promote new spaces for scientific research under different configurations or contexts due to the scarce national literature on the subject and the presence of difficulties and/or technical complexities in the area of study.

### 6.1. Implications

Our findings contribute to the wealth of literature by indicating that it is more likely that certain structural factors (including demographic, social, familial and cultural), rather than behavioral and medical factors, are associated with the level of perception of the modes of transmission and evolution of HIV/AIDS and by confirming that there is a marked difference in this knowledge at the national level. In the evaluation of the results, there are distinct factors that offer the possibility of modifications with the purpose of designing and implementing preventive programs under a structural approach.

Furthermore, by examining whether parametric and non-parametric algorithms could learn from existing national data and aid in the prediction of the knowledge about HIV/AIDS, the study shows that such an approach is feasible and that the algorithms achieved accuracy levels similar to those found in the literature review for predicting the perception and understanding of the dynamics and nature of the epidemic. This approach allowed us to establish the most predictive features for the knowledge by considering the complex nature of various predictors of HIV/AIDS understanding to provide an intuitive understanding of the key features. Similarly, this study demonstrates the potential of machine learning models to identify adolescents and young adults who have a greater exposure or predisposition to accept incorrect notions about HIV/AIDS transmission and engage in risky behaviors under those ideas based on certain regressors. In addition, this methodology facilitates the selection of a model based on resource-constrained performance and stability of performance over time. Incorporating such information into the algorithms can potentially improve the discriminative properties of the models. By experimenting with the design and execution of these machine learning algorithms, is clear that such models can identify relationships even when some of the input data are very complex, poorly defined or structured.

### 6.2. Limitations and Recommendations

In a general framework, the restricted availability of data on HIV/AIDS in Peru is a serious limitation in this study. The nature of the available data, to a large extent, adapted the direction of this thesis. The aggregation of the data at the national level made it impossible to perform an analysis at more detailed or specific levels. In addition, the study and modeling of the epidemic trend in the different demographic strata of Peruvian society were hampered by the lack of stratification of the data. It is recommended that, in the medium and long term, the data be expanded in favor of greater availability for the public domain after removing all patient identities and be more accessible to researchers.

In a different perspective, in computational terms, the use of the software established in the study presented difficulties for the development of the proposed models. In the case of RStudio (in particular, the *survey* package), it was challenging to correctly identify and use the proper complex survey designs in the study; it was difficult to includw non-response bias into the analyses; and when dealing with enormous datasets, it can be resource-intensive, and users may need access to sophisticated computational resources to operate successfully. For the software RapidMiner, preliminarily, the program has several features and functions that might be unfamiliar without adequate training; as processes become larger and more sophisticated, the workflows may become more complicated and harder to manage, making it difficult to discover and address issues; importing data from particular formats (Excel or CSV files) may pose a problem, needing data pre-processing or conversion prior to analysis; and when working with huge datasets or performing complicated analyses, it may be resource-intensive, requiring strong computational resources. Future research should consider employing solutions in programming languages that are more compatible with the programming skills of the researchers and with greater possibilities for scalability and efficient design.

On the other hand, it can be established that a limitation in the analysis is the static and associative measurement of knowledge about the epidemic and the socioeconomic, health and family factors inherent to the cross-sectional design. Future research should include longitudinal designs that can measure an individual’s current level of knowledge as well as future behavior to confirm the temporal and causal relationship. This type of data would allow for a convincing assessment of the effects of risk perception and knowledge on behavior.

Similarly, the use of data from secondary sources (such as the DHS survey) generated drawbacks that translate into the fact that the selection of variables, data quality and measurement indicators were beyond the control or determination of the researcher a priori. Similarly, the study may have had response biases during the collection of perceived risk or sensitive factors (i.e., performance of HIV/AIDS testing, self-perceived ethnicity, among others), although this concern is common to most studies of self-reported behavior. In addition, the data collected were from 2019; meanwhile, the knowledge of the target population may have changed and the results presented previously may not accurately reflect the present situation of knowledge among adolescents and young adults in Peru. Therefore, the patterns found in this study should be evaluated by health experts and researchers (who have expertise in the problem domain) to decide whether they are logical, practical and novel to drive new directions of biological and clinical research. Additionally, the use of additional covariates and perhaps sexual history data collected from different samples is recommended to draw further inferences about differences associated with community or individual characteristics. The identification of such differences could improve our understanding of the variation in HIV/AIDS knowledge observed in the population.

In another perspective, additional future studies are needed to further evaluate the usefulness and effects of the parametric and nonparametric algorithms employed and should be aimed at improving them. This may include the exploration of other machine learning techniques and configurations to improve model performance: variation in the complexity and dimensionality associated with model building, models that cater to the time-series nature of the problem, redefinition or inclusion of new predictors and exploration of underlying interactions in current models that can be exploited. In turn, noting the interpretability and comprehensibility deficiency from which certain machine learning methods employed in this section still suffer (e.g., if a machine learning method is exploiting some interaction and nonlinearity effects, the model-based examination of the importance of variables cannot fully explain and account for such predictive mechanisms), improving the interpretability mechanism in generating results is a critical and attractive, but largely understudied, direction for further research.

## Figures and Tables

**Table 1:** Operational definition of the socio-demographic, economic and family factors used in the study.

Variable	Definition	Scale	Type	Categories
HIV/AIDS knowledge	Knowledge about HIV/AIDS	Nominal	Dependent	0. NO
				1. YES
Gender	Gender of the respondent	Nominal	Independent	0. Female
				1. Male
Area of residence	Geographical setting of the interviewee	Nominal	Independent	0. Rural
				1. Urban
Educational level	Highest educational level achieved	Ordinal	Independent	0. Illiterate
				1. Elementary school
				2. High school
				3. Higher education
Marital status	Civil status of each individual	Nominal	Independent	0. Single
				1. Married/Cohabitant
				2. Divorced/Widowed/Separated
Nationality	Status of belonging to a particular nation	Nominal	Independent	0. Foreigner
				1. Peruvian
Ethnicity	Ethnicity that the respondent identifies with	Nominal	Independent	0. Native ${}^{1}$
				1. Afro-peruvian
				2. Caucasian
				3. Mixed
				4. Other/Does not know
Primary language	Main language the respondent learned in their first years of life	Nominal	Independent	0. Native language ${}^{2}$
				1. Spanish
				2. Foreign language
Heard about HIV/AIDS	Heard information about HIV/AIDS in the past	Nominal	Independent	0. NO
				1. YES
HIV/AIDS screening test	HIV/AIDS discard test performed in the last 12 months	Nominal	Independent	0. NO
				1. YES
Age	Respondent’s age range	Ordinal	Independent	0. 15 to 20
				1. 20 to 24
				2. 25 to 29
Region of residence	Region of residence to which the individual belongs	Nominal	Independent	0. Lima ${}^{3}$
				1. Coast
				2. Highlands
				3. Jungle
Mass media access	Ability to access mass media ${}^{4}$	Nominal	Independent	0. NO
				1. YES
Gender of household head	Gender of the head of household in the respondent’s home	Nominal	Independent	0. Female
				1. Male
Wealth index	Wealth group to which the respondent belongs	Ordinal	Independent	0. Lowest index
				1. Second lowest index
				2. Middle index
				3. Second highest index
				4. Highest index

^1^ Quechua, Aymara, native of the Amazon or part of another indigenous ethnicity. ^2^ Quechua or Aymara/native language of the jungle or other native languages. ^3^ It includes the province of Lima and the Constitutional Province of Callao. ^4^ Mass media includes access to radio, television or internet.

**Table 2:** Definition and types of hyper-parameters for parametric and nonparametric models.

Model	Hyper-Parameter	Type	Definition
L.R.	-	-	-
A.N.N.	Number of hidden layers	Integer	Describes the number of hidden layers within the network
	Number of neurons	Integer	Describes the number of neurons within the hidden layers
	Training cycles	Integer	Specifies the number of cycles used for training the network
	Learning rate	Real	Defines the cost of the gradient in updating a weight
	Momentum	Real	Adds a fraction of the previous weight update to the current one
k-NN	k number of neighbors	Integer	Describes the number of nearest neighbors to include in the process
	Weighted voting	Nominal	Determines whether distance values are involved in the prediction
	Types of measure	Nominal	Describes the measure chosen to find the nearest neighbors
R.F.	Number of trees	Integer	Specifies the number of random trees to generate
	Splitting criterion	Nominal	Selects the criterion on which attributes will be selected for splitting
	Maximum depth	Integer	Restricts the depth for each random tree
	Voting strategy	Nominal	Specifies the prediction strategy in case of dissenting tree model predictions
	Minimum gain	Real	Describes the threshold gain at a node before splitting it
	Minimum leaf size	Integer	Determines the minimum number of leaves to split an internal node
	Size for splitting	Integer	Determines the minimum size of an internal node for splitting
	Pre-prune alternatives	Integer	Sets the number of alternative nodes tested for splitting
D.T.	Split criterion	Nominal	Defines the criterion on which attributes will be chosen for splitting
	Maximum depth	Integer	Restricts the depth for each random tree
	Confidence level	Real	Specifies the level for the pessimistic pruning error calculation
	Minimum gain	Real	Describes the threshold gain at a node before splitting it
	Minimum leaf size	Integer	Determines the minimum number of leaves to split an internal node
	Size for splitting	Integer	Determines the minimum size of an internal node for splitting
	Pre-prune alternatives	Integer	Sets the number of alternative nodes tested for splitting

L.R.: Logistic regression, A.N.N.: Artificial neural networks, k-NN: k-nearest neighbors algorithm, R.F.: Random forest, D.T.: Decision tree.

**Table 3:** Statistical analysis of socio-demographic, economic and health factors according to the knowledge about HIV/AIDS.

	Inadequate Knowledge		Adequate Knowledge		
	(n = 6989)		(n = 3576)		
**Variable**	**Sample n** ${}^{1}$	**Weighted n** ${}^{2}$	**Sample n** ${}^{1}$	**Weighted n** ${}^{2}$	$\chi^{2}$ **Test**
**Gender**					*p* < 0.01 ***
Female	4142 (63.90%)	30.79% (0.0067)	2343 (36.10%)	19.97% (0.0061)	
Male	2847 (69.80%)	33.30% (0.0076)	1233 (30.20%)	15.94% (0.0063)	
**Area of residence**					*p* < 0.01 ***
Rural	2605 (76.60%)	13.73% (0.0036)	795 (23.40%)	4.28% (0.0023)	
Urban	4384 (61.20%)	50.36% (0.0081)	2781 (38.80%)	31.63% (0.0076)	
**Wealth index**					*p* < 0.01 ***
Lowest index	2534 (78.60%)	14.36% (0.0041)	691 (21.40%)	3.91% (0.0022)	
Second lowest index	2062 (66.10%)	17.16% (0.0056)	1058 (33.90%)	8.17 (0.0039)	
Middle index	1252 (60.70%)	14.11% (0.0057)	809 (39.30%)	8.97% (0.0047)	
Second highest index	732 (53.50%)	10.94% (0.0055)	636 (46.50%)	8.49% (0.0045)	
Highest index	409 (51.70%)	7.54% (0.0048)	382 (48.30%)	6.38% (0.0045)	
**Region of residence**					*p* < 0.01 ***
Lima	697 (58.60%)	21.74% (0.0082)	492 (41.40%)	15.42% (0.0068)	
Coast	1859 (61.00%)	16.41% (0.0051)	1191 (39.00%)	9.62% (0.0040)	
Highlands	2566 (72.00%)	16.66% (0.0054)	996 (28.00%)	6.77% (0.0030)	
Jungle	1867 (67.50%)	9.28% (0.0037)	897 (32.50%)	4.10% (0.0021)	
**Age**					*p* < 0.01 ***
15 to 20	2072 (71.70%)	22.91% (0.0069)	817 (28.30%)	10.32% (0.0051)	
21 to 24	2182 (65.20%)	21.66% (0.0063)	1164 (34.80%)	12.22% (0.0052)	
25 to 29	2735 (63.20%)	19.52% (0.0054)	1595 (36.80%)	13.37% (0.0048)	
**Educational level**					*p* < 0.01 ***
Illiterate	29 (93.50%)	0.17% (0.0005)	2 (6.50%)	0.01% (0.0001)	
Elementary school	924 (87.40%)	5.52% (0.0028)	133 (12.60%)	9.93% (0.0013)	
High school	4413 (69.70%)	40.80% (0.0078)	1920 (30.30%)	19.53% (0.0062)	
Higher education	1623 (51.60%)	17.61% (0.0062)	1521 (48.40%)	15.37% (0.0056)	
**Ethnicity**					*p* < 0.01 ***
Native	2520 (70.40%)	17.39% (0.0055)	1059 (29.60%)	8.38% (0.0043)	
Afro-peruvian	868 (73.00%)	8.88% (0.0041)	321 (27.00%)	3.38% (0.0027)	
Caucasian	466 (70.20%)	4.88% (0.0031)	198 (29.80%)	2.07% (0.0022)	
Mixed	2568 (58.70%)	27.53% (0.0071)	1805 (41.30%)	19.99% (0.0067)	
Other/Does not know	567 (74.60%)	5.41% (0.0037)	193 (25.40%)	2.08% (0.0026)	
**Heard about HIV/AIDS**					*p* < 0.01 ***
NO	1182 (84.60%)	7.84% (0.0035)	215 (15.40%)	1.66% (0.0018)	
YES	5807 (63.30%)	56.25% (0.0075)	3361 (36.70%)	34.25% (0.0076)	
**HIV/AIDS screening test**					*p* < 0.01 ***
NO	5338 (68.30%)	50.41% (0.0077)	2473 (31.70%)	26.22% (0.0070)	
YES	1651 (59.90%)	13.68% (0.0049)	1103 (40.10%)	9.69% (0.0044)	
**Marital status**					0.499 N.S.
Single	2930 (66.10%)	33.95% (0.0075)	1501 (33.90%)	18.54% (0.0065)	
Married/Cohabitant	3603 (66.50%)	26.71% (0.0062)	1811 (33.50%)	15.19% (0.0053)	
Divorced/Widowed/Separated	456 (63.30%)	3.44% (0.0026)	264 (36.70%)	2.17% (0.0021)	
**Mass media access**					*p* < 0.01 ***
NO	865 (77.60%)	5.29% (0.0029)	250 (22.40%)	1.72% (0.0078)	
YES	6124 (64.80%)	58.80% (0.0018)	3326 (35.20%)	34.19% (0.0075)	
**Gender of household head**					p=0.014 **
Female	1861 (63.50%)	17.40% (0.0055)	1068 (36.50%)	11.00% (0.0048)	
Male	5128 (67.20%)	46.69% (0.0075)	2508 (32.80%)	24.90% (0.0070)	
**Primary language**					*p* < 0.01 ***
Native language	1447 (77.90%)	7.76% (0.0034)	411 (22.10%)	2.59% (0.0023)	
Spanish	5533 (63.60%)	56.25% (0.0079)	3164 (36.40%)	33.31% (0.0075)	
Foreign language	9(90.00%)	0.09% (0.0004)	1 (10.00%)	0.01% (0.0001)	
**Nationality**					*p* = 0.275 N.S.
Foreigner	76 (59.40%)	0.02% (0.0021)	52 (40.60%)	0.017% (0.0019)	
Peruvian	6913 (66.20%)	65.77% (0.0078)	3523 (33.80%)	34.193% (0.0041)	

** very significant values *p* < 0.05; *** highly significant values *p* < 0.01. N.S.: Not statistically significant. ^1^ Frequency and percentage of unweighted observations are calculated. ^2^ Proportions and standard error of weighted observations are calculated.

**Table 4:** Results of the multivariate analysis of the association between socio-demographic, economic and health factors and knowledge about HIV/AIDS.

Variable	β	Std. Error	*t*	*p*-Value	O.R.a (I.C. 95%)	GVIF ${}^{1}$
**Intercept**	−3.595	0.921	−3.903	*p* < 0.01 ***	0.03 (0.00–0.17)	-
**Gender**						1.028
Female (REF)	-	-	-	-	-	
Male	−0.334	0.070	−4.751	*p* < 0.01 ***	0.72 (0.62–0.82)	
**Wealth index**						1.170
Lowest index (REF)	-	-	-	-	-	
Second lowest index	0.298	0.112	2.649	*p* = 0.008 ***	1.35 (1.08–1.68)	
Middle index	0.467	0.135	3.466	*p* < 0.01 ***	1.60 (1.22–2.08)	
Second highest index	0.558	0.145	3.843	*p* < 0.01 ***	1.75 (1.31–2.32)	
Highest index	0.617	0.163	3.774	*p* < 0.01 ***	1.85 (1.35–2.55)	
**Area of residence**						1.431
Rural (REF)	-	-	-	-	-	
Urban	−0.076	0.093	−0.815	*p* = 0.415 N.S.	0.93 (0.77–1.11)	
**Region of residence**						1.121
Lima (REF)	-	-	-	-	-	
Coast	−0.019	0.093	−0.204	*p* = 0.838 N.S.	0.98 (0.82–1.18)	
Highlands	−0.178	0.101	−1.761	*p* = 0.078 *	0.84 (0.69–1.02)	
Jungle	−0.030	0.106	−0.284	*p* = 0.777 N.S.	0.97 (0.79–1.19)	
**Age**						1.083
15 to 20 (REF)	-	-	-	-	-	
21 to 24	0.089	0.090	0.977	*p* = 0.329 N.S.	1.09 (0.92–1.30)	
25 to 29	0.285	0.094	3.020	*p* = 0.003 ***	1.33 (1.11–1.60)	
**Educational level**						1.097
Illiterate (REF)	-	-	-	-	-	
Elementary school	1.249	0.909	1.375	*p* = 0.169 N.S.	3.49 (0.59–20.69)	
High school	1.911	0.894	2.137	*p* = 0.033 **	6.76 (1.17–38.98)	
Higher education	2.198	0.894	2.457	*p* = 0.014 ***	9.00 (1.56–51.96)	
**Ethnicity**						1.086
Native (REF)	-	-	-	-	-	
Afro-peruvian	−0.328	0.131	−2.502	*p* = 0.012 **	0.72 (0.56–0.93)	
Caucasian	−0.207	0.152	−1.364	*p* = 0.173 N.S.	0.81 (0.60–1.09)	
Mixed	0.092	0.098	0.933	*p* = 0.351 N.S.	1.10 (0.90–1.33)	
Other/Does not know	−0.387	0.160	−2.420	*p* = 0.016 **	0.68 (0.50–0.93)	
**Heard about HIV/AIDS**						1.067
NO (REF)	-	-	-	-	-	
YES	0.635	0.127	4.992	*p* < 0.01 ***	1.89 (1.47–2.42)	
**HIV/AIDS screening test**						1.040
NO (REF)	-	-	-	-	-	
YES	0.174	0.074	2.352	*p* = 0.019 **	1.19 (1.03–1.38)	
**Mass media access**						1.116
NO (REF)	-	-	-	-	-	
YES	0.063	0.131	0.476	*p* = 0.634 N.S.	1.06 (0.82–1.38)	
**Gender of household head**						1.060
Female (REF)	-	-	-	-	-	
Male	−0.091	0.075	−1.220	*p* = 0.223 N.S.	0.91 (0.79–1.06)	
**Primary language**						1.130
Native language (REF)	-	-	-	-	-	
Spanish	0.233	0.120	1.942	*p* = 0.052 *	1.26 (1.00–1.60)	
Foreign language	−1.915	1.102	−1.738	*p* = 0.082 *	0.15 (0.02–1.28)	

* Significant values *p* < 0.10; ** very significant values *p* < 0.05; *** highly significant values *p* < 0.01; N.S.: Not statistically significant. REF: Factor reference level. ^1^ Generalized Variance Inflation Factor.

**Table 5:** Hyper-parameter testing and selection of optimal values for parametric and non-parametric models.

Hyper-Parameter	Available Choice	Selected Choice ${}^{1}$
	**A.N.N.**	
Number of hidden layers	1	**1**
Number of neurons	10–20–30–40–50–60–70–80–90–100	**20**
Training cycles	50–60–70–80–90–100–110–120–130–140–150	**70**
Learning rate	0.0001–0.001–0.01–0.1	**0.1**
Momentum	0.5–0.6–0.7–0.8–0.9	**0.9**
	**k-NN**	
k number of neighbors	2–3–4–5–6	**3**
Weighted vote	YES–NO	**NO**
Types of measurement	Mixed Measures–Numerical measures	**Mixed Measures (M.E.D.) ${}^{2}$**
	**R.F.**	
Number of trees	10–20–30–40–50–60–70–80–90–100	**40**
Division criterion	Information gain–Gain ratio	**Information gain**
Maximum depth	5–10–15–20	**15**
Voting strategy	Confidence vote–Majority vote	**Majority vote**
Minimum gain	0.001–0.01–0.1	**0.01**
Minimum leaf size	1–2–3–4–5–6	**1**
Size for division	1–2–3–4–5–6	**4**
Pre-prunning alternatives	1–2–3–4–5–6	**1**
	**D.T.**	
Division criteria	Information gain–Gain ratio	**Information gain**
Maximum depth	5–10–15–20	**15**
Confidence level	0.1–0.2–0.3–0.4–0.5	**0.4**
Minimum gain	0.001–0.01–0.1	**0.01**
Minimum leaf size	1–2–3–4–5–6	**5**
Size for division	1–2–3–4–5–6	**5**
Pre-pruning alternatives	1–2–3–4–5–6	**5**

A.N.N.: Artificial neural networks, k-NN: k-nearest neighbors algorithm, R.F.: Random forest, D.T.: Decision tree. ^1^ The optimal hyper-parameters considered and evaluated were those that complied with the condition of not generating fit problems (overfitting or underfitting) within the model training and that generated the best goodness-of-fit results. ^2^ M.E.D.: Mixed Euclidean Distance.

**Table 6:** Performance metric comparison of parametric and non-parametric models.

Model	L.R.	A.N.N.	k-NN	R.F.	D.T.
**Metrics**	%/n	%/n	%/n	%/n	%/n
TP	177	149	78	180 *	136
TN	475	526	550 *	499	540
FP	223	172	148 *	199	158
FN	181	209	280	178 *	222
FPR	31.95	26.64	21.20 *	28.51	22.64
FNR	50.56	58.38	78.21	49.72 *	62.01
Accuracy	61.74	63.92	59.47	64.30 *	64.02
Sensitivity/TPR	49.44	41.62	21.79	50.28 *	37.99
Specificity/TNR	68.05	75.36	78.80 *	71.49	77.36
Cohen’s kappa ${}^{1}$	0.170	0.174	0.006	0.215 *	0.161
F1-Score	46.70	43.89	26.71	48.85 *	41.72
AUC	62.90 *	62.90 *	48.90	61.20	60.20

L.R.: Logistic regression, A.N.N.: Artificial neural network, k-NN: k-nearest neighbors algorithm, R.F.: Random forest, D.T.: Decision Tree. ^1^ The result is shown in absolute terms. *: Best value according to performance metric.

## Data Availability

The datasets used and/or analyzed during the current study are available from the corresponding author upon reasonable request.

## Abbreviations

The following abbreviations are used in this manuscript:

HIV	Human Immunodeficiency Virus
AIDS	Acquired Immunodeficiency Syndrome
ART	Antiretroviral Therapy
DHS	Demographic and Family Health Survey
GVIF	Generalized Variance Inflation Factor
